# Genetic diversity of genus *Chilomastix*: molecular classification of *C. mesnili* and other potential species variations in humans and animals

**DOI:** 10.1186/s41182-025-00725-5

**Published:** 2025-03-27

**Authors:** Chuanhao Jiang, Siti Arifah Lacante, Tetsushi Mizuno, Din Syafruddin, Masaharu Tokoro

**Affiliations:** 1 Department of Global Infectious Diseases, Graduate School of Advanced Preventive Medical Sciences, Kanazawa University, 13-1 Takaramachi, Kanazawa, Ishikawa 920-8640 Japan; 2 Graduate School of Hasanuddin University, Jl. Perintis Kemerdekaan Km.10, Makassar, Sulawesi Selatan Indonesia; 3 Department of Parasitology, Faculty of Medicine, Hasanuddin University, Jl. Perintis Kemerdekaan Km.10, Makassar, Sulawesi Selatan Indonesia; 4 Department of Global Infectious Diseases, Graduate School of Medical Sciences, Kanazawa University, 13-1 Takaramachi, Kanazawa, Ishikawa Japan

**Keywords:** Genus *Chilomastix*, *Chilomastix mesnili*, Genetic diversity, Molecular taxonomy

## Abstract

**Background:**

The genus *Chilomastix*, including *C. mesnili*, consists of protozoa that parasitize the gastrointestinal tracts of various host organisms, including mammals (humans and non-human primates [NHP]), birds, and amphibians. Despite its widespread presence, *Chilomastix* spp. are generally considered non-pathogenic, which has led to limited molecular epidemiological studies on this genus. Consequently, genetic reference data for this genus remain scarce in GenBank. In this study, we aimed to establish a molecular classification for *Chilomastix* spp. by investigating the genetic diversity of isolates from humans and animals in a parasite-endemic region of Indonesia.

**Methods:**

A cross-sectional molecular investigation was conducted in Wainyapu Village, Sumba Island, Indonesia. Stool samples were collected annually from 2013 to 2016 and screened using polymerase chain reaction (PCR) targeting the 18S small subunit ribosomal RNA gene (18S rRNA) of *Chilomastix* spp., followed by direct and subcloning sequencing. Genetic haplotypes of the partial 18S rRNA sequence (1386–1953 bp) from humans (*n* = 25), dogs (*n* = 1), pigs (*n* = 23), rats (*n* = 38), water buffaloes (*n* = 3), chickens (*n* = 10), and ducks (*n* = 1) were analyzed alongside reference sequences from humans, guinea pigs, leeches, frogs, and water sources using phylogenetic analyses.

**Results:**

The prevalence of *Chilomastix* spp. was 7.0% (25/356) in humans and 19.7% (75/380) in animals. Phylogenetic analyses revealed the following monophyletic clusters as subtypes (STs): *C. mesnili* ST1 (human–NHP genotype), *C. mesnili* ST2-1 (human genotype), and *C. mesnili* ST2-2 (pig genotype). In addition, *C. gallinarum*-like haplotypes (chicken genotype) and *C. bettencourti*-like haplotypes, including ST1 (rat genotype) and ST2 (rat–buffalo genotype), were also identified.

**Conclusions:**

The genetic references registered in this study, along with the revealed molecular classification of *Chilomastix* spp., are crucial for understanding the genetic diversity and host-specific dynamics of these parasites in endemic regions.

**Supplementary Information:**

The online version contains supplementary material available at 10.1186/s41182-025-00725-5.

## Background

The formal species name *Chilomastix mesnili* has a complex history. The earliest documented record of this intestinal protozoan likely dates back to 1854 in Paris, when it was detected in a patient with diarrhea and described by Davaine as *Cercomonas hominis* var. 1 [1, 2]. In 1910, this human intestinal protozoan parasite was renamed *Macrostoma mesnili* by Wenyon [3]. Subsequently, in 1920, Kofoid reorganized the naming of flagellates and renamed it *Chilomastix davainei* (syn. *Chilomastix mesnili)* [1, 4]; however, genus *Chilomastix* was previously used by Alexeieff in 1912 for another flagellate belonging to the same genus [1, 4]. As a result, Wenyon's species name and Alexeieff's genus name were combined, leading to the formal name *Chilomastix mesnili* (Wenyon, 1910) Alexeieff, 1912.

Although there have been some exceptional clinical cases, such as a Japanese traveler to China and India [5], an elderly Chinese man [6], and a prolonged diarrheal case in Nderu, Kenya [7], *C. mesnili*, the only known *Chilomastix* sp. detected in humans, has generally been considered a harmless commensal protozoan. It may only be of concern in patients with compromised immune systems, such as in AIDS patients [5, 8] as a cause of diarrheal disease. Similarly, there have been a few case reports of *Chilomastix* spp. in other host species, such as a case of enteritis in horses by *Chilomastix equi* [9, 10], and a watery diarrheal case in birds by *Chilomastix gallinarum* [11].

Host species of *Chilomastix* spp. include various vertebrates (mammals, birds, and amphibians) and insects, such as cockroaches [12, 13], while a single known free-living species, *Chilomastix cuspidata* has also reported [14]. Currently, more than 30 species of genus *Chilomastix* have been described based on morphological characteristics and host specificity [11]. However, regarding genetic information from the 18S small subunit ribosomal RNA (18S rRNA) gene locus, only 21 partial sequences representing four *Chilomastix* species (*C. mesnili*, *C. cuspidata*, *C. caulleryi, and C. wenrich*) are available in GenBank. Recent efforts to establish a taxonomic system for protists based on molecular classification have placed the genus *Chilomastix* within the family Retortamonadidae, under the phylum Metamonada [15]. Nevertheless, the monophyly of Retortamonadidae remains unverified, as some studies suggest that the clade could be polyphyletic [16]. This ambiguity persists due to the limited availability of genetic reference data. To resolve this classification uncertainty and establish a molecular framework for *Chilomastix* spp., we conducted a molecular evaluation using samples obtained from humans and various animal hosts in Indonesia, an endemic area for this parasite.

## Methods

### Sample collection and DNA extraction

**Fig. 1:**
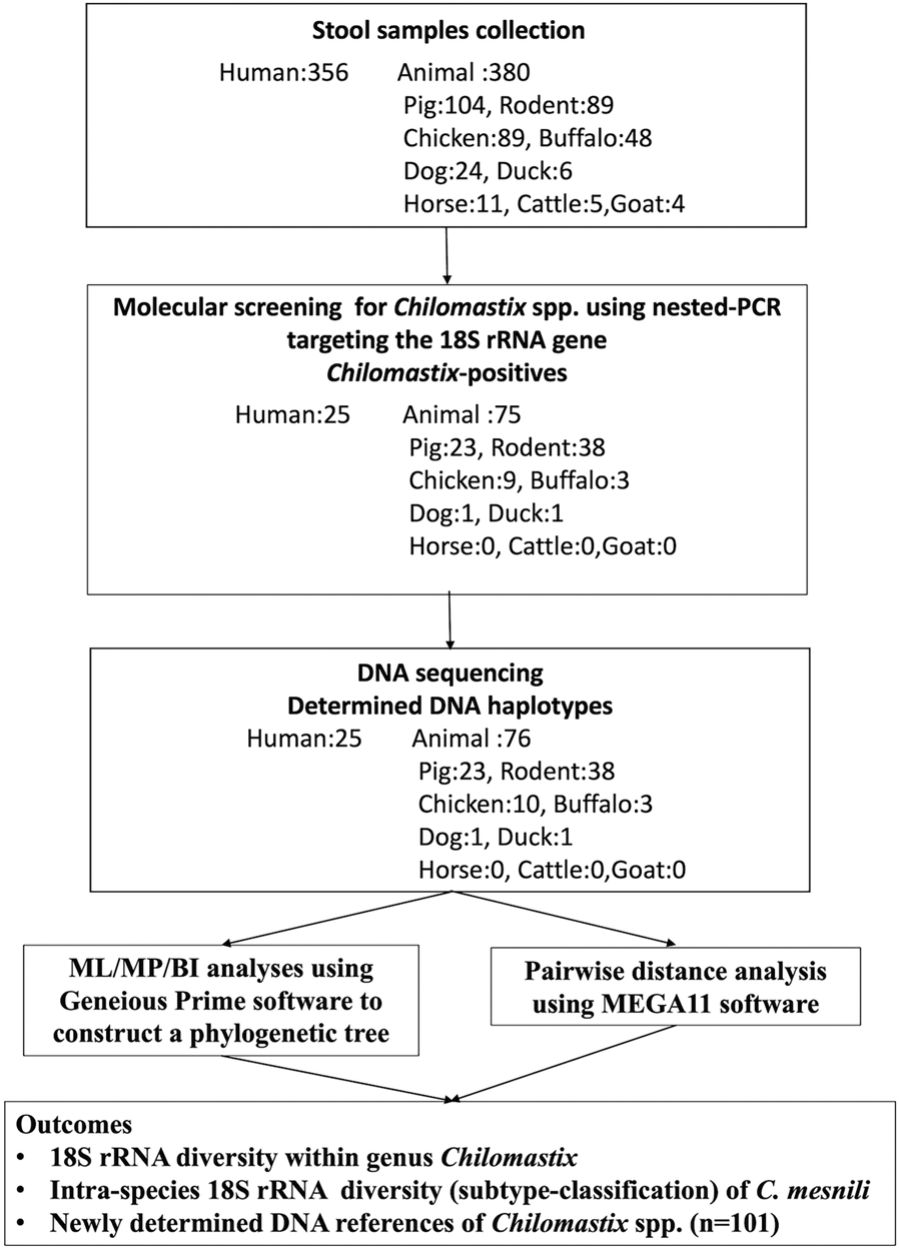
Research flowchart of this study

The overall workflow of this study is summarized in the research flowchart (Fig. 1). Stool samples were collected in Wainyapu village, Sumba Island, Indonesia. Human stool samples were obtained from healthy school children (aged 7–14) at Kera-Panba Primary and Junior High School (9° 38′ 36.13″ S and 119° 0′ 58.05″ E, respectively). The study area is located in an equatorial tropical climate zone. The local population is primarily engaged in agriculture and maintains a close daily relationship with livestock and companion animals, which were evaluated in this study. Field sample collection was conducted annually during the dry season in this region, from August to September, between 2013 and 2016. Animal stool samples were collected from various animals across the village, including pigs (*n* = 104), rats (*n* = 89), chickens (*n* = 89), buffaloes (*n* = 48), dogs (*n* = 24), horses (*n* = 11), ducks (*n* = 6), cattle (*n* = 5), and goats (*n* = 4).

Stool sample treatment was performed as previously described [17]. Briefly, all stool samples were collected from healthy individuals using the following methods: for humans, stool bags were distributed, and the discharged stools were collected at schools. For buffaloes, goats, and horses, stool samples were collected immediately after discharge on the ground. For pigs and dogs, stool samples were collected using an enema. For rats, chickens, and ducks, stool samples were collected directly from the intestinal tract by dissection. From each collected sample, 0.2 g of stool was preserved in 600 µL of DNAzol® reagent (Molecular Research Center, Inc., Cincinnati, OH, USA) in 1.5 mL screw-capped tubes. The DNAzol-treated stool samples were stored at room temperature (~ 25 °C) for up to 1 week at the field site, followed by storage at 4 °C in the laboratory until further analysis. Genomic DNA was extracted from the DNAzol®-preserved samples following the manufacturer’s protocol with modifications. Samples underwent two freeze–thaw cycles between − 80 °C and room temperature, followed by overnight digestion with proteinase K (0.4 mg/mL; Wako Pure Chemical Industries, Osaka, Japan) at 55 °C. DNA was precipitated using ethanol and suspended in 30 µL of 10 mM Tris–HCl (pH 8.0) containing 1 mM EDTA. Extracted DNA was stored at − 20 °C until further analysis.

### Polymerase chain reaction (PCR) and DNA sequencing

Nested PCR was performed to amplify a partial region of the 18S rRNA gene. The first PCR used a universal primer set for intestinal protozoa (TN21′: 5′-AAGATTAAGCCATGCATG-3′/TN14′: 5′-ACCTTGTTACGACTTCTCCTT-3′), which we previously designed and utilized [17]. The second PCR employed a newly designed *Chilomastix* spp.-specific primer set (TN117: 5′-TGCTAATACGTGCACCWAATG-3′/MT846: 5′-GACCATACTCCCCCCGT-3′), based on the alignment of available reference sequences of *Chilomastix* spp. in GenBank: *C. mesnili* (EU009463, EU009464, EU009465, EU009466, KC960583, KC960584, KC960585, KC960586, KC960587, KC960588), *C. caulleryi* (AB600326), *C. cuspidata* (KC960591), and *Chilomastix* sp.-IC-2014 (KC960593).

PCR reactions (10 µL) were performed using LA-Taq polymerase with GC buffer (TaKaRa Bio Inc., Shiga, Japan) in a MyCycler thermal cycler (Bio-Rad Laboratories, California, USA). The reaction mixture contained 0.5% dimethyl sulfoxide, with template volumes of 1 µL and 0.5 µL for the first and second PCRs, respectively. The cycling conditions for the primary PCR consisted of an initial denaturation at 94 °C for 1 min, followed by 25 cycles of 94 °C for 30 s, 50 °C for 30 s, and 72 °C for 3 min, with a final extension at 72 °C for 3 min. The secondary PCR was performed under the same conditions, except that the annealing temperature was set to 52 °C and the extension step was reduced to 2 min.

PCR products were purified using the FastGene® Gel/PCR Extraction Kit (Nippon Genetics, Tokyo, Japan). Direct sequencing was performed using the ABI Prism Big Dye v3.1 Cycle Sequencing Kit (Life Technologies Japan, Tokyo, Japan) or the SupreDye™ Cycle Sequencing Kit v3.1 (AdvancedSeq LLC, CA, USA) on an Applied Biosystems 3130 Genetic Analyzer (Life Technologies Japan). Sequencing was confirmed using forward and reverse primers for the second PCR, with additional primers designed based on the obtained sequences and applied as necessary for read extension.

Due to sequence heterogeneity or difficulties encountered during direct sequencing, nine chicken-derived PCR amplicons required subcloning procedures for sequence determination. The amplicons were cloned into the pMD20-T plasmid using the Takara TA-Mighty Cloning Kit (Takara Bio Inc., Japan), transformed into *Escherichia coli* DH5-α, and screened on LB agar with 100 mg/L ampicillin. Positive colonies carrying the subcloned plasmids were cultured in LB liquid medium with 100 mg/L ampicillin, and the plasmids were extracted using the NucleoSpin® Plasmid QuickPure Kit (Macherey–Nagel GmbH & Co. KG, Germany). The inserted DNA fragments were sequenced using the above-mentioned sequencing method.

All confirmed sequences of the partial 18S rRNA gene fragment of *Chilomastix* spp. from this study have been deposited in the DNA Database (DDBJ–EMBL–GenBank) under accession numbers LC789656–LC789756 (*n* = 101).

### Data analysis

Phylogenetic reconstructions were performed using maximum likelihood (ML) and maximum parsimony (MP) methods in Geneious Prime 2023.2.1 (Biomatters Ltd., Auckland, New Zealand). Bayesian inference (BI) was conducted using the MrBayes 3.2.6 extension in the same software [18]. Clade support was assessed based on bootstrap proportions (BP) > 50% for ML and MP, as well as posterior probability (PP) values > 0.5 for BI. Pairwise genetic distances were calculated using MEGA11 [19].

### Limitation of research

This study was performed as a molecular epidemiological investigation utilizing a stock of fecal samples collected over several years from residents of a rural village in Indonesia and domesticated animals living in close contact with humans. As the samples were obtained through multiple cross-sectional collections over an extended period, they were collected opportunistically rather than systematically. Therefore, the positivity rates and other numerical values were not considered epidemiologically significant. In addition, the findings were based on geographically limited samples. Furthermore, the 18S rRNA gene region targeted in the PCR analysis was a partial rather than a full-length sequence. The overlap with available reference sequences was also limited, which may affect the resolution of the phylogenetic analysis. Compared to evaluations based on full-length sequences or genomic data, the phylogenetic resolution in this study is expected to be relatively low.

## Results

### Summary of molecular screening

Molecular screening using our specific nested PCR protocol targeting *Chilomastix* spp. infection confirmed its prevalence in 7.0% (25/356) of human samples, while it was 19.7% (75/380) in total animal samples (Table 1). The prevalence in each animal species was as follows: pig (23/104, 22.1%), rat (38/89, 42.7%), chicken (9/89, 10.1%), buffalo (3/48, 6.3%), dog (1/24, 4.2%), and duck (1/6, 16.7%). No positive samples were found in horses, goats, or cattle. For each positive sample, a single DNA haplotype of the partial 18S rRNA sequence was typically identified. Meanwhile, in nine chicken samples subjected to subcloning, two haplotypes were detected within a single sample, resulting in a total of 10 haplotypes identified across 9 samples. As a result of this molecular screening, a total of 101 partial 18S rRNA sequences of *Chilomastix* spp. were identified in this study and subsequently deposited in the genetic database.

**Table 1:** Summary of molecular screening results of *Chilomastix* spp. from human and animal stool samples in Wainyapu village, Sumba Island, Indonesia

Host	Total	*Chilomastix* spp. positives (%)	Registered DDBJ accession numbers ^*^	Sequence length (bp)
Human *Homo sapiens*	356	25 (7.0)	LC789661–LC789671, LC789684, LC789685, LC789699–LC789702, LC789716–LC789723	1386–1459
Animal	380	75 (19.7)		
Pig *Sus scrofa domesticus*	104	23 (22.1)	LC789672–LC789678, LC789686–LC789689, LC789724–LC789735	1406–1948
Rodent *Rattus exulans*	89	38 (42.7)	LC789679–LC789683, LC789690–LC789698, LC789703–LC789705, LC789736–LC789756	1556–1953
Chicken *Gallus gallus*	89	9 (10.1)	LC789659, LC789706–LC789714	1453–1821
Buffalo *Bubalus bubalis* ^**^	48	3 (6.3)	LC789656–LC789658	1461–1558
Dog *Canis lupus familiaris*	24	1 (4.2)	LC789660	1453
Duck *Anas platyrhynchos*	6	1 (16.7)	LC789715	1558

^*^Partial sequence of 18S rRNA gene, ^**^domestic water buffalo

### Phylogenetic placement of *Chilomastix* spp. within Fornicata

**Fig. 2:**
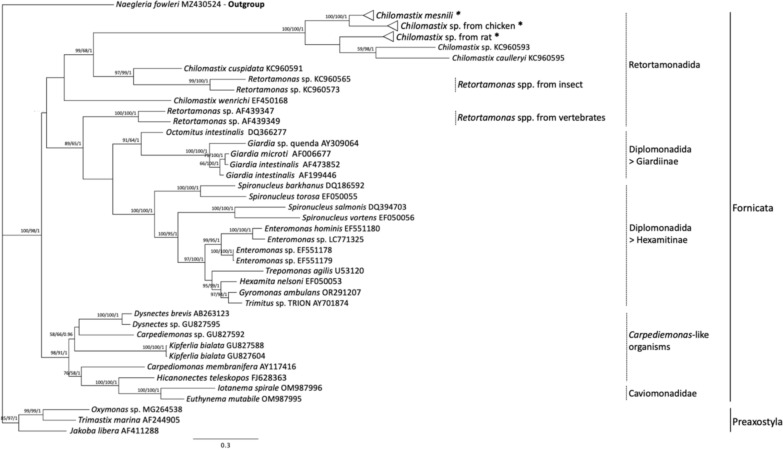
Genetic diversity of Diplomonadida, *Carpediemonas*-like organisms, Caviomonadidae, and Retortamonadida among Fornicata. The totally 210 nucleotide positions were used as the final data set. The best tree of BI analysis with the HKY85 model is shown with the PP value along with the BP values for the ML and MP analyses. The order of numerical sources is ML/MP/BI. The asterisk indicates the cluster that contains haplotypes from this study

To confirm that the DNA haplotypes detected through molecular screening in this study definitively belong to *Chilomastix* spp. and to exclude the possibility of cross-amplification of non-targeted species, we performed phylogenetic analyses using ML, MP, and PI methods. These analyses incorporated reference partial 18S rRNA gene sequences from genera closely related to *Chilomastix* in Fornicata, along with all DNA haplotypes identified as *Chilomastix* spp. in this study (Fig. 2). The reference data set comprised sequences from four *Chilomastix* species: *C. caulleryi* (KC960595; derived from a frog), *Chilomastix* sp. (KC960593; derived from a leech), *C. cuspidata* (KC960591; derived from an environmental sample), and *C. wenrichi* (EF450168; derived from a guinea pig). In addition, it included sequences from 30 other species in Diplomonadida, Carpediemonas-like organisms, Caviomonadidae, and Retortamonadida, all of which are classified under Fornicata, as well as three reference sequences belonging to Preaxostyla, a closely related group to Fornicata. *Naegleria fowleri* was also used as an outgroup.

All DNA haplotypes identified in this study formed a statistically well-supported monophyletic clade, which included the reference sequences of *C. mesnili*, *C. caulleryi*, and *Chilomastix* sp. (KC960593) (ML: BP = 100%, MP: BP = 100%, BI: PP = 1.0). This main *Chilomastix* spp. clade was positioned as a sister clade to a distinct monophyletic clade comprising *C. cuspidata* and the insect-derived *Retortamonas* sp. (KC960565, KC960573). Furthermore, the parent clade, which includes both the main *Chilomastix* spp. clade and the monophyletic clade of *C. cuspidata* and *Retortamonas* sp., was well-supported as monophyletic (ML: BP = 99%, MP: BP = 68%, BI: PP = 1.0). Interestingly, *C. wenrichi* (EF450168) was positioned as an outgroup to the parent clade. Furthermore, its monophyly with the parent clade, which includes all other *Chilomastix* spp., was not statistically supported.

**Fig. 3:**
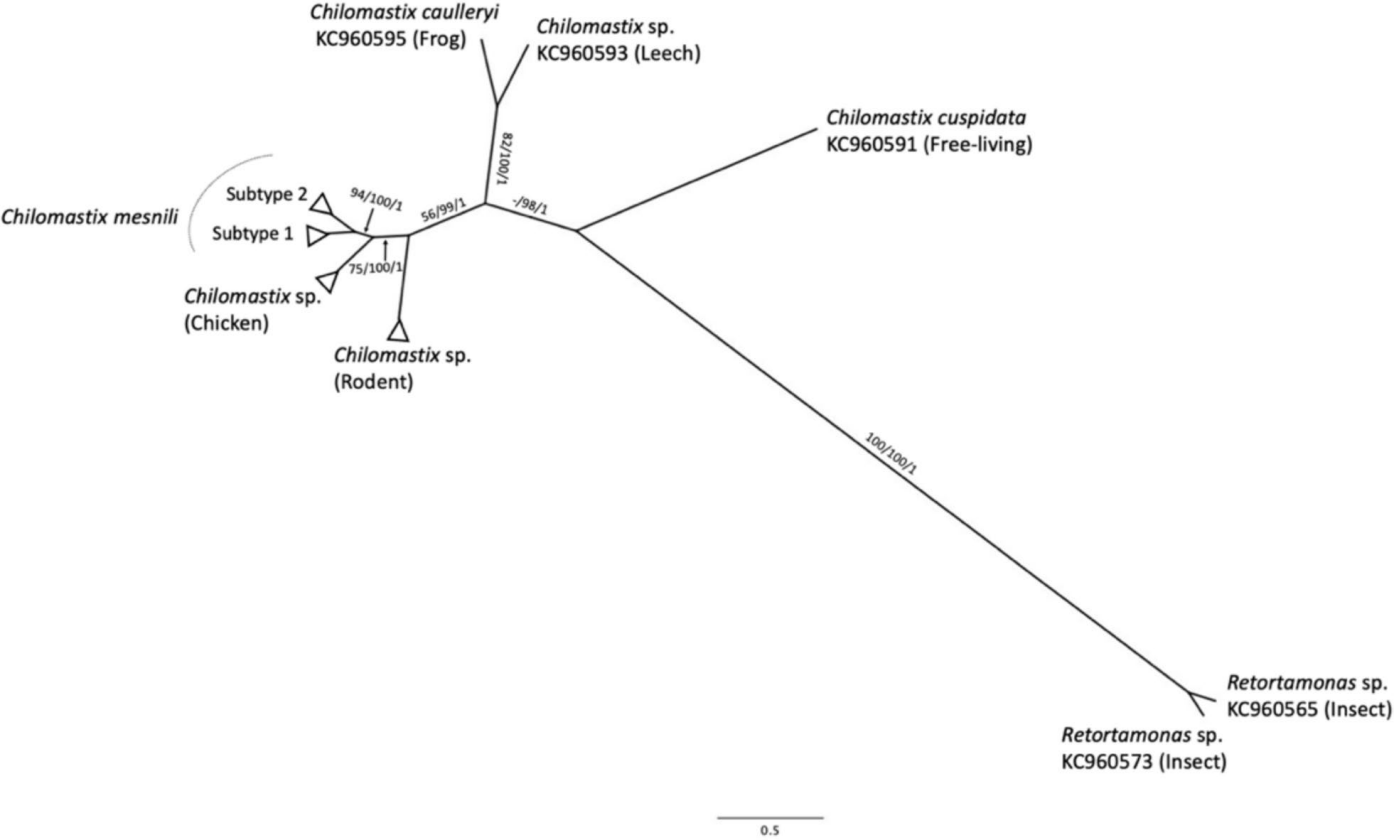
Overview of phylogenetic relationships among *Chilomastix* spp. and *Retortamonads* spp. The totally 738 nucleotide positions were used as the final data set. The best tree of BI analysis with the HKY85 model is shown with the PP value along with the BP values for the ML and MP analyses. The order of numerical sources is ML/MP/BI. Subtypes were defined as the clusters formed by the first branching within *C. mesnili* clade

Since most of the reference sequences used in the analysis were partial and corresponded to different regions of the 18S rRNA gene locus, the final data set for the phylogenetic analysis included only 210 nucleotide positions. To extend this region and enhance phylogenetic resolution, we conducted an additional phylogenetic analysis using a longer analyzable DNA sequence, which included 738 nucleotide positions, by employing a subset of sequences restricted to those within the parent clade containing *Chilomastix* spp. and *Retortamonas* sp. (Fig. 3). This analysis revealed the presence of three statistically significant clades. The first of these clades (ML: BP = 56%, MP: BP = 99%, BI: PP = 1.0) comprised a cluster of *C. mesnili* and two independent clusters of *Chilomastix* sp., mainly derived from chickens and rats, respectively. The second clade (ML: BP = 82%, MP: BP = 100%, BI: PP = 1.0) consisted of the amphibian-derived *C. caulleryi* and the leech-derived *Chilomastix* sp. Finally, the third clade (ML: BP = –, MP: BP = 98%, BI: PP = 1.0) included *C. cuspidata* and the insect-derived *Retortamonas* sp.

### Genetic diversity of genus *Chilomastix*

Although the employed sequence regions including only conserved 738 nucleotide positions, all haplotype variations of the DNA sequences confirmed in this study were primarily clustered within the main *Chilomastix* spp. clade (Fig. 3).

To further clarify the genetic classification of the detected sequences, we incorporated longer reference sequences for comparison. Based on sequence alignment including 1151 conserved nucleotide positions, a total of 33 unique haplotypes were identified from the 101 sequences confirmed in this study (Table 2).

**Table 2:** Summary of DNA haplotypes identified in this study

Subtype classification	Host 1*	Host 2	Host 3	GC contents	Sequence length (bp) **	DDBJ accession number ***
*C. mesnili* ST1	P7_2014			55.2%	1406	LC789686
*C. mesnili* ST1	*Chilomastix mesnili* EU009464 (human)			55.1%	1394	EU009464
*C. mesnili* ST1	H211_H702_H1288_H3026_H3190_2013, H925_2016			55.0%	1389	LC789661
*C. mesnili* ST1	H3122_2013			54.9%	1389	LC789668
*C. mesnili* ST1	H728_H4025_H4331_2016			54.9%	1389	LC789717
*C. mesnili* ST1	H242_2013, H211_2015, HA008_2016			54.9%	1387	LC789662
*C. mesnili* ST1	H798_2014, H798_2016			54.9%	1386	LC789684
*C. mesnili* ST1	*Chilomastix mesnili* EU009466 (human)			54.8%	1407	EU009466
*C. mesnili* ST2-1	*Chilomastix mesnili* KC960588 (human)			57.0%	1448	KC960588
*C. mesnili* ST2-1	*Chilomastix mesnili* KC960587 (human)			56.9%	1447	KC960587
*C. mesnili* ST2-1	H3020_H3138_HA008_2013, HA008_2014, H60_H164_HA008_2015, H10_2016			56.8%	1441	LC789666
*C. mesnili* ST2-1	H1289_2013			56.7%	1435	LC789665
*C. mesnili* ST2-2	P27_2013			56.3%	1460	LC789678
*C. mesnili* ST2-2	P7_P8_2016			56.3%	1459	LC789729
*C. mesnili* ST2-2	P7_2013			56.3%	1446	LC789673
*C. mesnili* ST2-2	P23_P25_2013, P2_P3_P10_P11_P14_2016			56.2%	1460	LC789676
*C. mesnili* ST2-2	P8_2014			56.2%	1455	LC789687
*C. mesnili* ST2-2	P21_2013			56.2%	1454	LC789675
*C. mesnili* ST2-2	P9_P10_2014, P4_P9_2016	Ch34_2013	D9_2013	56.2%	1453	LC789688
*C. mesnili* ST2-2	H3112_2016			56.1%	1459	LC789720
*C. mesnili* ST2-2	P1_P13_2013, P5_P15_2016			56.0%	1461	LC789672
*C. mesnili* ST2-2	B13_2013			56.0%	1461	LC789657
rat-derived *Chilomastix* sp. ST1	R18_2016			51.5%	1951	LC789741
rat-derived *Chilomastix* sp. ST1	R6_R8_R12_2013, R4_R15_R23_2014, R27_2016	P1_2016		51.3%	1948	LC789681
rat-derived *Chilomastix* sp. ST1	R6_2014			51.3%	1948	LC789691
rat-derived *Chilomastix* sp. ST1	R17_2014			51.3%	1947	LC789695
rat-derived *Chilomastix* sp. ST1	R1_2013, R6_R8_2015			51.2%	1950	LC789679
rat-derived *Chilomastix* sp. ST1	R5_2015			51.2%	1950	LC789703
rat-derived *Chilomastix* sp. ST1	R16_2016			51.2%	1949	LC789740
rat-derived *Chilomastix* sp. ST1	R32_2016			51.1%	1953	LC789750
rat-derived *Chilomastix* sp. ST1	R16_2014			51.0%	1944	LC789694
rat-derived *Chilomastix* sp. ST2	R14_2014			49.6%	1562	LC789692
rat-derived *Chilomastix* sp. ST2	R4_2013, R21_R22_2014, R10_R11_R12_R13_R19_R21_R23_R24_R25_R26_R28_R39_2016			49.6%	1560	LC789680
rat-derived *Chilomastix* sp. ST2	B12_B21_2013	Duck2_2016		49.6%	1558	LC789656
rat-derived *Chilomastix* sp. ST2	R34_R35_R37_R41_R42_2016			49.6%	1556	LC789751
chicken-derived *Chilomastix* sp.	Ch1_Ch2_Ch3_Ch4_Ch6.2_Ch11_2016			62.8%	1821	LC789706
chicken-derived *Chilomastix* sp.	Ch5_Ch6.1_Ch8_2016			62.3%	1607	LC789710

^*^Hosts 1, 2, and 3 show the detected host of each haplotype (H: human; P: pig; B: buffalo; Ch: chicken; D: dog; R: rat; and Duck: duck), sample ID, and the years of sampling (2013, 2014, 2015, and 2016)

^**^All sequences including references were compared based on the excised region, which corresponds to 1,151 conserved nucleotide positions within the total length of the original sequences

^***^A representative accession number is provided in cases where multiple samples share the same haplotype

**Fig. 4:**
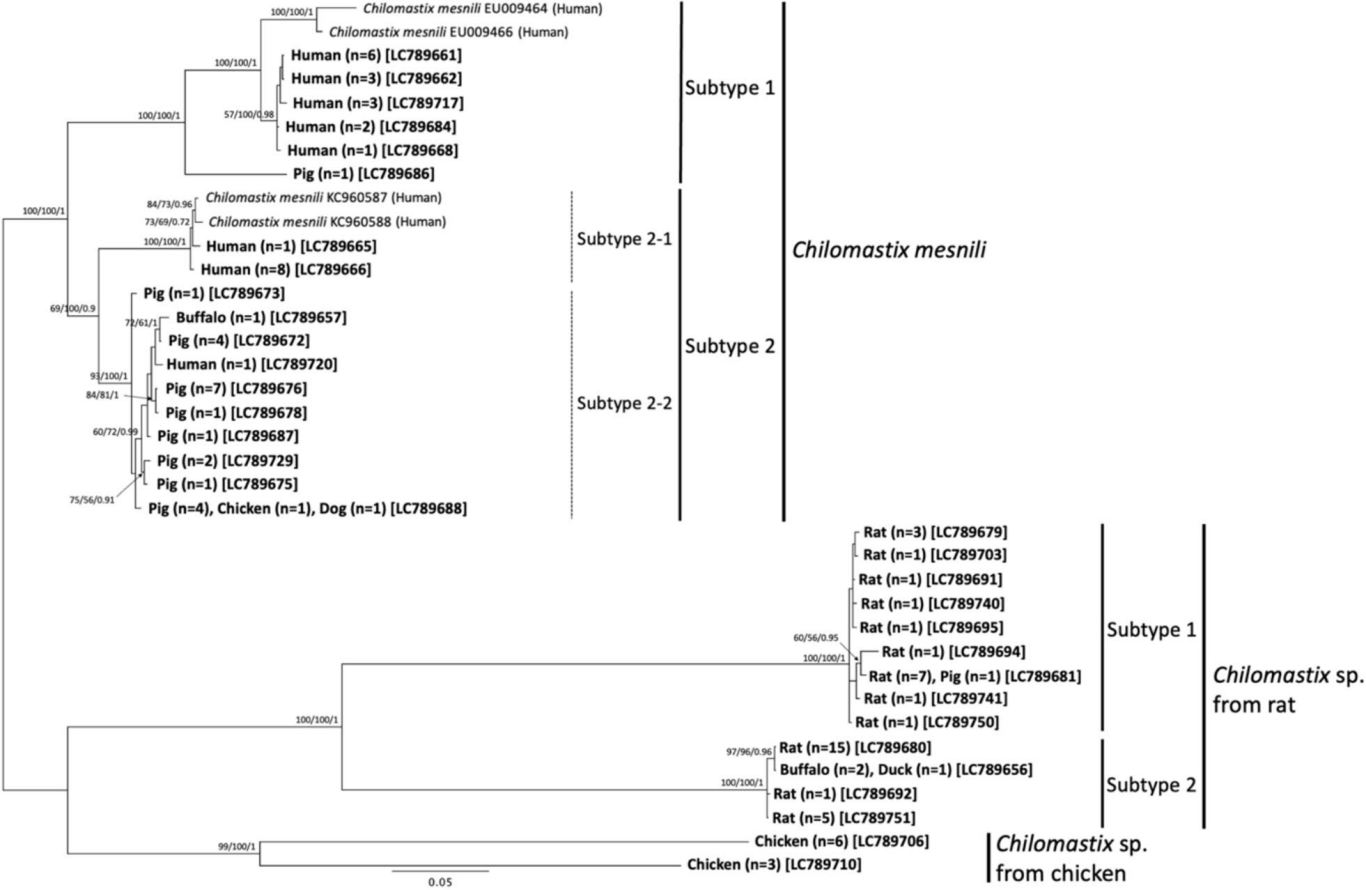
Genetic diversity of *Chilomastix* spp. Phylogenetic tree inferred using unduplicated partial 18S rRNA sequences, including 37 haplotypes, of which 33 were confirmed in this study and 4 were reference sequences. A total of 1,151 nucleotide positions were used as the final data set. The best tree from the BI analysis, constructed using the HKY85 model, is shown with PP values alongside BP values from the ML and MP analyses. The numerical order of the support values is ML/MP/BI. The numbers in the tip labels indicate the number of samples for each host assigned to the designated haplotype. Square brackets indicate the accession number. The list of all haplotypes is provided in Table 2

The phylogenetic analysis of the main *Chilomastix* spp. clade, including the 33 unique haplotypes and four *C. mesnili* reference sequences, revealed three monophyletic groups (Fig. 4).

The first group (ML: BP = 100%, MP: BP = 100%, BI: PP = 1.0) included all reference sequences of *C. mesnili* and was, therefore, designated as the *C. mesnili* clade. Within this clade, two statistically well-supported sister clusters were identified, which we propose as subtype (ST) classifications: ST1 (ML: BP = 100%, MP: BP = 100%, BI: PP = 1.0) and ST2 (ML: BP = 69%, MP: BP = 100%, BI: PP = 0.9). For reference, a subtype was defined as the cluster formed by the first branching within each potential species clade, provided that it contained two or more haplotypes. Furthermore, within each subtype, the cluster formed by the first branching was considered an additional variation within the subtype. The ST2 cluster of the *C. mesnili* clade contained two statistically significant sub-clusters, designated ST2-1 (ML: BP = 100%, MP: BP = 100%, BI: PP = 1.0) and ST2-2 (ML: BP = 93%, MP: BP = 100%, BI: PP = 1.0). The included DNA haplotypes of the partial 18S rRNA gene region of each *C. mesnili* ST exhibited variations in both sequence length and GC content, as follows (Table 2): *C. mesnili* ST1 (1386–1407 bp; GC content: 54.8–55.2%), ST2-1 (1435–1448 bp; GC content: 56.7–57.0%), and ST2-2 (1446–1461 bp; GC content: 56.0–56.3%).

Within the main *Chilomastix* spp. clade, the second group (ML: BP = 100%, MP: BP = 100%, BI: PP = 1.0) and the third group (ML: BP = 99%, MP: BP = 100%, BI: PP = 1.0) were primarily composed of haplotypes detected from rats and chickens, respectively, and were designated as the rat-derived *Chilomastix* sp. clade and the chicken-derived *Chilomastix* sp. clade. The rat-derived *Chilomastix* sp. clade comprised two statistically well-supported sister clusters, designated ST1 (ML: BP = 100%, MP: BP = 100%, BI: PP = 1.0) and ST2 (ML: BP = 100%, MP: BP = 100%, BI: PP = 1.0) within this clade. The DNA haplotype variation in both sequence length and GC content within these monophyletic clusters was as follows: rat-derived *Chilomastix* sp. ST1 (1,944–1,953 bp; GC content: 51.0–51.5%), rat-derived *Chilomastix* sp. ST2 (1556–1562 bp; GC content: 49.6%), and chicken-derived *Chilomastix* sp. (1607 bp, 1821 bp; GC content: 62.3%, 62.8%).

Regarding host specificity, the following findings were confirmed (Table 2). In the *C. mesnili* clade, ST1 was detected in humans and pigs, whereas ST2 was identified in humans, pigs, dogs, buffaloes, and chickens. Within ST2, sub-clusters exhibited different host ranges: ST2-1 was found only in humans, while ST2-2 was mainly detected in pigs, with each single detection in humans, buffaloes, chickens, and dogs. In the rat-derived *Chilomastix* sp. clade, ST1 was mainly detected in rats, with a single detection in pigs. Similarly, ST2 was mainly detected in rats but was also found in two buffaloes and one duck. In the chicken-derived *Chilomastix* sp. cluster, all detections were from chickens.

## Discussion

The family Retortamonadida consists of two genera, *Retortamonas* spp. and *Chilomastix* spp.; however, the monophyly of Retortamonadida remains uncertain. Previous studies have suggested that vertebrate-derived *Retortamonas* spp. might be better placed within Diplomonadida rather than Retortamonadida [16, 20, 21].

Our previous phylogenetic analyses, which included *Retortamonas* spp. detected from a wide range of vertebrate hosts, placed vertebrate-derived *Retortamonas* spp. within Diplomonadida. In contrast, insect-derived *Retortamonas* spp. formed a distinct cluster together with *C. mesnili*, *C. caulleryi*, *C. cuspidata*, and *C. wenrichi*. These findings suggest that the monophyletic *Retortamonas* spp. cluster, which served as the basis for the family name Retortamonadida, may not actually exist within this taxonomic family [22].

In this study, we further assessed the taxonomic placement of *Retortamonas* spp. and *Chilomastix* spp. by incorporating new reference sequences of *Chilomastix* spp. Our analysis revealed that insect-derived *Retortamonas* spp. formed a distinct monophyletic cluster with *C. cuspidata*, which was positioned as a sister cluster to the main *Chilomastix* spp. clade (Fig. 2). Moreover, *C. wenrichi* was positioned as an outgroup to the parent clade of these sister groups. Compared to previous studies that placed insect-derived *Retortamonas* spp. in the same cluster as *Chilomastix* spp., the present results provide a more detailed phylogenetic perspective. The findings suggest that genus classifications require revision not only for insect-derived *Retortamonas* spp. but also for *C. cuspidata* and possibly *C. wenrichi* as well. However, a more detailed phylogenetic analysis using 738 nucleotide positions (Fig. 3) showed that the genetic divergence between insect-derived *Retortamonas* spp. and *C. cuspidata* was greater than that between the *C. mesnili* main clade and *C. cuspidata*. Therefore, the revision of genus classifications will require longer DNA sequences for more robust assessments.

A genotyping classification based on the genetic diversity of *Chilomastix* spp. has rarely been attempted due to the extremely limited availability of genetic references for this genus, including *C. mesnili*. A previous study proposed a classification of *C. mesnili* into two genetic clusters, cluster A and cluster B. This classification was based on the analysis of two DNA haplotypes of *C. mesnili* detected in Japanese macaques along with 12 human-derived reference sequences. In this classification, Cluster A was designated as the human genotype, while Cluster B included sequences from both humans and Japanese macaques [23]. However, since the reference sequence from the Japanese macaque was relatively short (878 bp of the 18S rRNA gene), those reference sequences were not included in the phylogenetic analysis of this study. Nevertheless, when comparing the reference sequences from the previous study, the Cluster B including the Japanese macaques haplotypes corresponds to *C. mesnili* ST1 as proposed in this study, whereas other subtypes were not identified in the previous study.

In this study, 33 novel unique haplotypes were incorporated into the phylogenetic analysis of *Chilomastix* spp. genetic diversity, leading to the identification of several monophyletic clusters and the proposal of a refined genotyping classification. Within the *C. mesnili* main clade, two monophyletic subtypes (ST1 and ST2) were proposed, with ST2 further subdivided into two sub-clusters, ST2-1 and ST2-2.

This study utilized PCR-based screening of *Chilomastix* spp. in fecal samples, which inherently does not confirm actual infections. The possibility of detecting DNA from uninfected *Chilomastix* cysts that were mechanically passed through the gut after accidental ingestion cannot be ruled out. To account for this limitation, a fundamental criterion was applied for host identification: a host species was not considered a definitive host if only a single sample from that species was detected within a monophyletic group. This criterion allows for future reassessment should additional samples be detected in subsequent studies.

Applying this criterion and incorporating previous findings from Japanese macaques as non-human primates (NHP), the host specificity of each ST in *C. mesnili* can be summarized as follows: *C. mesnili* ST1 (human–NHP genotype), *C. mesnili* ST2 (human–pig genotype), *C. mesnili* ST2-1 (human genotype), and *C. mesnili* ST2-2 (pig genotype).

In our phylogenetic analysis (Fig. 4), two additional monophyletic clades were identified outside the *C. mesnili* clade. The haplotypes in one clade were primarily detected in chickens, while those in the other clade were mainly detected in rats. Currently, no DNA reference sequences are available that belong to these clades. However, *C. gallinarum* [24] from chickens and *C. bettencourti* [25] from rats have been described based on morphological identification. Therefore, we designated the *Chilomastix* sp. primarily detected in chickens as *C. gallinarum*-like haplotypes and the *Chilomastix* sp. primarily detected in rats as *C. bettencourti*-like haplotypes.

Applying the host-specificity criterion, these clades were described as *C. gallinarum*-like haplotypes (chicken genotype) and *C. bettencourti*-like haplotypes (rat–buffalo genotype). Furthermore, within the *C. bettencourti*-like haplotype clade, two sister clades were identified and designated as ST1 (rat genotype) and ST2 (rat–buffalo genotype).

Several features of these haplotypes suggest that these clades represent distinct *Chilomastix* species rather than intraspecific variants of *C. mesnili*. The phylogenetic tree showed relatively long branch lengths for these clades compared to the intraspecific diversity observed within the *C. mesnili* clade, indicating greater genetic divergence. Sequence homology analysis of the 18S rRNA gene further supported this distinction (Supplementary Table 1). The sequence identity between *C. mesnili* ST1 and *C. gallinarum*-like haplotypes ranged from 80.6 to 82.8%, while that between *C. mesnili* ST1 and *C. bettencourti*-like haplotypes ranged from 78.8 to 79.3%. These values were substantially lower than the sequence similarity observed between *C. mesnili* ST1 and ST2 (89.3–91.6%), reinforcing the hypothesis that *C. gallinarum*-like and *C. bettencourti*-like haplotypes belong to separate species.

In addition, differences in sequence length and base composition further supported this distinction. The *C. bettencourti*-like haplotypes exhibited considerable variation in sequence length and GC content, with ST1 ranging from 1944 to 1953 bp (GC content: 51.0–51.5%) and ST2 ranging from 1556 to 1562 bp (GC content: 49.6%). Similarly, the *C. gallinarum*-like haplotypes were 1821 bp and 1607 bp in length, with GC contents of 62.3% and 62.8%, respectively. In contrast, the *C. mesnili* clade exhibited a significantly shorter sequence length (1386–1461 bp) and a distinct GC content range (54.8–57.0%). These substantial genetic differences further supported the hypothesis that the *C. gallinarum*-like and *C. bettencourti*-like haplotypes represent distinct *Chilomastix* species rather than intraspecific variants of *C. mesnili*.

This study was conducted as part of a series of investigations aimed at elucidating the prevalence and genetic diversity of intestinal protozoa in humans and animals in parasite-endemic regions of developing countries. In the 2016 human samples (*n* = 144), in addition to the 5.6% (8/144) prevalence of *C. mesnili* detected in this study, our previous evaluation had revealed high prevalence rates of various intestinal protozoa as follows: *Giardia intestinalis* 56.3% (81/144), *Entamoeba histolytica* 0% (0/144), *E. dispar* 6.9% (10/144), *E. hartmanni* 31.3% (45/144), *E. coli* 44.4% (64/144), and *Enteromonas* sp. 38.2% (55/144) [17, 26]. In addition, our assessment of samples from the same region in 2013 (*n* = 290) had identified *Retortamonas* sp. in 4 cases (1.4%) [22].

Notably, the highly endemic protozoa in the study site were all commensals except for *G. intestinalis*; however, our previous findings indicated no significant correlation between giardial colonization and diarrheal stool form in this endemic setting [26]. Moreover, a haplotype classified as *C. mesnili* ST2-1 [LC789666], which was identified in multiple human samples (H3020_H3138_HA008_2013, HA008_2014, H60_H164_HA008_2015, H10_2016), was detected continuously over 3 years (from 2013 to 2015) in an individual child, HA008 (Table 2).

Such persistent and chronic protozoal parasitization without harmful reactions suggests that these protozoa may constitute part of the gut microbiota, forming a stable "protozoal flora" similar to the bacterial flora. To appropriately assess the role of such diverse commensal intestinal protozoa as a functional community within the host gut (protozoal flora), comprehensive metagenomic analyses, similar to those applied to gut bacteria, are essential for understanding their ecological interactions and contributions to the gut microbiota. Therefore, for numerous non-pathogenic intestinal protozoa that previously had only limited genetic reference data available, it is necessary to establish a framework for detailed molecular classification and register reference sequences, as demonstrated in this study.

## Conclusions

This study provides the first 18S rRNA gene-based reference for *Chilomastix* spp. in several animal hosts, including pigs, rats, chickens, buffaloes, dogs, and ducks. A total of 101 genetic references, including 33 unique DNA haplotypes, were registered in this study. Based on the phylogenetic analysis, the identified monophyletic clades were utilized to propose the molecular classification of *Chilomastix* spp. as follows: *C. mesnili* ST1 (human–NHP genotype), *C. mesnili* ST2-1 (human genotype), and *C. mesnili* ST2-2 (pig genotype). In addition, *C. gallinarum*-like haplotypes (chicken genotype) and *C. bettencourti*-like haplotypes, including ST1 (rat genotype) and ST2 (rat–buffalo genotype), were also proposed. These findings are crucial for understanding the genetic diversity and host-specific dynamics of these parasites in endemic regions.

## Supplementary Information

Supplementary Material 1. Table 1. Genetic distances among the confirmed haplotypes in this study

## Data Availability

DNA sequences of the 18S rRNA gene locus have been deposited into the DNA Database (DDBJ-EMBL-Genbank) data library with accession number LC789656-LC789756.

## Abbreviations

18S rRNA: 18S small subunit ribosomal RNA
BI: Bayesian Inference
EDTA: Ethylenediaminetetraacetic acid
ML: Maximum likelihood
MP: Maximum parsimony
PCR: Polymerase chain reaction
PP: Posterior probability
BP: Bootstrap proportion
ST: Subtype
NHP: Non-human primate
